# The effects of thermotherapy and cryotherapy on pain and fear during intravenous cannulation: a systematic review and meta-analysis

**DOI:** 10.7586/jkbns.25.039

**Published:** 2025-08-28

**Authors:** Jihoo Her, Ki-Yong Kim, Yu-Jin Lee, Bo-Song Kim, Myung-Haeng Hur

**Affiliations:** 1 College of Nursing, Gimcheon University, Gimcheon, Korea; 2 College of Nursing, Eulji University, Gimcheon, Korea; 3 Daejeon Eulji University Hospital, Daejeon, Korea

**Keywords:** Cryotherapy, Thermotherapy, Pain, Fear, Injections, intravenous

## Abstract

**Purpose:**

Intravenous (IV) cannulation is a common hospital procedure that often causes pain and discomfort, leading to injection-related fear. Therefore, effective strategies for alleviating IV-related pain are necessary. To investigate the impact of thermotherapy and cryotherapy on pain and fear associated with IV cannulation, we reviewed randomized controlled trials.

**Methods:**

PubMed, Embase, Cochrane, CINAHL, Research Information Service System, DBpia, NSDL, and the Korean Studies Information Service System were searched for studies available in databases as of December 31, 2024. The study participants were individuals who underwent IV line (angio-needle) insertion. The intervention group received thermotherapy or cryotherapy for pain management, and the comparison group received no treatment or placebo. The outcome variables were pain and fear.

**Results:**

Significantly lower pain levels were observed in the thermotherapy and cryotherapy groups compared to the control group. The effect size of thermotherapy was shown by a standardized mean difference (SMD) of −0.68, while the SMD for cryotherapy was −0.93. No significant difference was found between thermotherapy and cryotherapy (*p* = .430). The effect of thermotherapy and cryotherapy on IV-related fear was also significant (SMD = −1.74; *p* = .005).

**Conclusion:**

This meta-analysis confirms that both thermotherapy and cryotherapy are effective in reducing IV-related pain and fear, with no significant difference between the two approaches. However, thermotherapy may offer additional benefits, such as reducing IV insertion time and improving patient satisfaction. These findings suggest that thermotherapy and cryotherapy are viable non-pharmacological options for IV-related pain and fear management.

## INTRODUCTION

Intravenous (IV) cannulation is one of the most common procedures performed for diagnostic or therapeutic purposes when patients visit or are admitted to a hospital. It is performed in approximately 60%~70% of hospitalized patients [1] and serves various purposes during hospitalization, such as blood sampling for tests, medication administration, continuous fluid therapy, blood transfusion, and preparation for emergencies [2-4]. Despite being a routine procedure, needle insertion often evokes pain and negative memories, especially from early-life experiences [5-7] compounded by the necessity to endure it for medical care. This pain represents a complex integration of physical, psychological, and social experiences and emotions [7,8]. Notably, younger children, older individuals, or those requiring repeated injections may experience persistent discomfort associated with needle insertion [5, 9-10].

Advancements in hospital-based treatment methods have led to significant progress in diagnostic and therapeutic procedures, including research aimed at alleviating pain during needle insertion. Needle-free injection systems, designed to administer medication without the use of needles, have been reported to cause less pain [11] and are particularly utilized in dental anesthesia [12]. However, while these systems are effective for intramuscular or intradermal injections, they are not yet applicable for IV cannulation, which still relies on conventional needle use.

IV cannulation often induces pain and discomfort, and fear of needles can lead some patients to refuse treatment [5,9,10]. As small-gauge needles (22~24G) are typically utilized in simple procedures such as blood sampling or venipuncture, these are less uncomfortable. Meanwhile, the insertion of an IV catheter for prolonged medication administration or blood transfusion can cause greater discomfort due to the complexity of the procedure, the insertion site, and the use of larger needles [13,14]. Such pain may lead to psychological issues, such as anxiety and fear, in patients [15] and increase the workload of the nurses. Ideally, IV cannulation with devices such as IV catheters should be successful with minimal attempts [16]; however, it is not feasible to guarantee this outcome in every procedure. Therefore, effective interventions to temporarily alleviate pain during IV insertion are necessary. These interventions are considered to not only contribute to the patient's emotional stability but also play an important role in improving nursing efficiency [17,18].

Various randomized controlled trials have applied methods such as virtual reality devices [19], vibratory stimulation [20], topical anesthetic creams [20,21], thermotherapy, and cryotherapy [22,23] to alleviate pain, anxiety, and fear during IV cannulation. Among these, cryotherapy and thermotherapy are independent nursing interventions that can be easily applied in clinical settings, and directly implement the pain control mechanisms described by the Gate Control Theory [24-26]. Unlike topical anesthetic creams, which may have delayed onset and potential side effects, thermotherapy and cryotherapy are safe, low-cost, and immediately applicable interventions that can be initiated by nurses without a physician’s order. In addition to their physiological effects, thermotherapy and cryotherapy may reduce fear by providing a soothing sensory stimulus, which distracts attention from the procedure and enhances the patient's sense of comfort and control [27]. Cryotherapy reduces blood flow and inhibits the release of neurotransmitters, inducing a local anesthetic effect to alleviate pain, while thermotherapy promotes vasodilation in both peripheral and systemic blood vessels, enhancing blood flow [25,28]. This increase in blood flow activates tissue metabolism, reduces vascular tension, and suppresses the terminal branches of the autonomic nervous system, ultimately decreasing pain [29].

The use of thermotherapy and cryotherapy for pain management during IV catheter insertion initially involved simple applications such as ice packs or hot packs [30]. Over time, these methods have evolved into various forms of thermal and cryotherapy applications. Specifically, the application of cryotherapy has advanced to include methods such as cold spray, vibration devices combined with cryotherapy (e.g., Buzzy^®^ device), or thermoelectric element devices providing thermotherapy or cryotherapy [31,32]. Thermotherapy and cryotherapy have not only been implemented in diverse ways but have also been assessed using various outcome measures including pain, stress, anxiety, fear, and blood flow velocity.

Systematic reviews and meta-analyses have examined whether thermotherapy and cryotherapy are effective methods for alleviating IV injection-related pain [33,34]. A previous meta-analysis evaluating vapor coolants (cold spray) reported that their application was effective in alleviating pain during IV cannulation [34,35]. Another study [33] demonstrated that these coolants were more effective for venous puncture than arterial puncture and were particularly beneficial for adults than for children. In a meta-analysis of studies using the Buzzy^®^ device, which combines vibration and cold stimuli, significant reductions in pain and anxiety were observed in pediatric patients undergoing venipuncture [34]. Similarly, meta-analyses of interventions combining vibration and cryotherapy reported significant effects [35]. However, when thermoelectric element devices were used to provide cryotherapy and thermotherapy, no significant differences in venipuncture pain and anxiety were observed [32]. On the other hand, cryotherapy applied in the form of a tourniquet resulted in significantly lower pain levels compared to those in the control group [31].

These therapies aim to alleviate pain as well as to reduce anxiety and fear during the procedure. However, the outcomes vary with intervention type and application method [33]. Theoretically, thermotherapy has the potential to alleviate pain; however, few meta-analyses have independently examined its effects.

There is a need for additional well-designed studies that explore the separate impacts of thermotherapy and cryotherapy on pain and fear during IV cannulation, contributing to evidence-based combined approaches This study aimed to systematically evaluate the independent effects of thermotherapy and cryotherapy on IV-related pain and fear.

## METHODS

To objectively evaluate the effectiveness of thermotherapy and cryotherapy for pain and fear management during IV catheter insertion, our study followed the process of literature searching and study selection in accordance with the guidelines developed by the Preferred Reporting Items for Systematic Reviews and Meta-Analyses (PRISMA) group [36], and was registered with PROSPERO (CRD42024567711). Additionally, as a meta-analysis utilizing previously published data, it was exempted from ethical review (GU-202407-HRc-05).

### 1. Inclusion and exclusion criteria

This study used the PICO-SD framework. P (Population): individuals who underwent IV line insertion; I (Intervention): thermotherapy and cryotherapy for pain management; C (Comparison): no treatment or placebo; O (Outcome): pain and fear; SD (Study Design): randomized controlled trials.

Studies published in Korean or English that focused on heat or cold therapy during venipuncture procedures were included into this study. Priority was given to studies measuring pain during venipuncture or immediately after the procedure, rather than before the intervention began or after it ended. Only studies involving IV line insertion with an angio needle were included, while those involving simple venipuncture procedures, defined as procedures for collecting blood specimens using a standard hypodermic needle without an angiocatheter, were excluded. Preclinical or animal studies, studies not published in Korean or English, studies without full texts, non-experimental designs, studies combining pharmacological interventions with heat or cold therapy, or studies combining heat or cold therapy with other interventions such as vibration therapy were excluded. We included in the qualitative synthesis all studies that reported outcome data in any form (e.g., median with range, categorical data, or mean ± standard deviation [SD]), and only those that provided values in mean ± SD were included in the quantitative synthesis.

### 2. Search strategy and data sources

The literature search encompassed studies published from the earliest available date in each database to December 31, 2024. Searches were performed using international databases such as PubMed, CINAHL, Cochrane, and EMBASE, alongside domestic databases including DBpia, NDSL, Korean Studies Information Service System, and Research Information Service System. To ensure comprehensive coverage, gray literature such as theses, news articles, and presentation materials was manually searched using Google Scholar.

The search strategy incorporated both MeSH terms and text words, employing AND/OR operators and truncation techniques. For participants, search terms included “venipuncture,” “injections,” “intravenous,” “IV,” “intravenous catheter,” “intravenous cannulation,” and “intravenous cannula.” For intervention, terms related to hot and cold therapy were used, such as “hot,” “warm,” “heat,” “ice,” “cold,” “vapor coolant,” “cryotherapy,” and “thermotherapy.” For outcome, terms such as “pain” and “fear” were utilized to identify relevant studies.

Considering the lack of MeSH functionality in some domestic databases, additional Korean terms were employed, including “venipuncture,” “intravenous access,” “IV,” and “fear.” This strategy ensured thorough identification of relevant studies across all selected databases (Appendix 1).

### 3. Data extraction

Bibliographic data were organized in Microsoft Excel. Duplicate records were removed based on title and author information. Titles and abstracts were independently screened by two researchers in the first round of screening following the specified criteria for inclusion and exclusion. The full texts of the studies that passed the first round of screening were then reviewed by the same researchers in the second round of screening, and reasons for exclusion (e.g., animal studies, language, study design, statistical data, interventions involving pharmacotherapy, or combined interventions involving non-thermal modalities) were recorded.

Studies that applied both thermotherapy and cryotherapy within a single group were excluded, while studies that applied them separately in independent groups were included.

Consistency at each stage was ensured through cross-checks, and disagreements between researchers were resolved through thorough discussion until a consensus was reached. Although a third reviewer was available to resolve any discrepancies, no disagreements occurred between the two researchers during data extraction.

**Figure 1.**
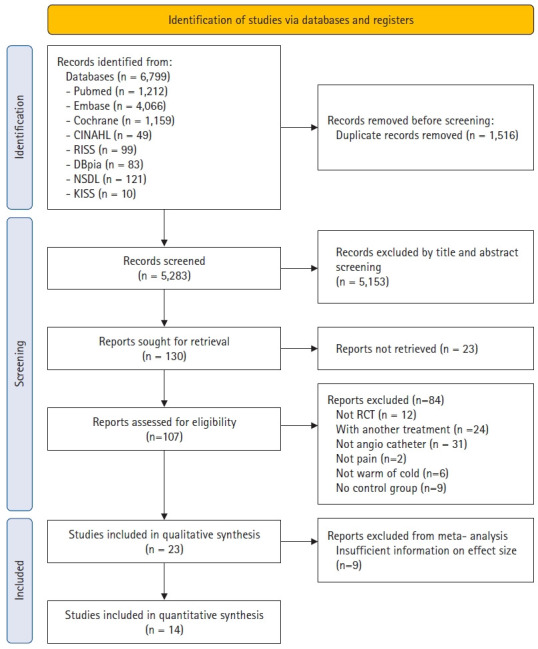
Flow chart of study selection process.

A coding book was developed to record general characteristics of the studies, including study participants, country of sampling, participant sex and age, sample size, and intervention details for experimental and control groups (e.g., type of intervention, duration of application). Outcome variables such as pain and fear scores were extracted for effect size analyses. Data coding was independently performed by two researchers, and discrepancies were resolved through a joint review of the original texts, to finalize the coding. The process of study selection and exclusion was visualized using a PRISMA flow diagram (Figure 1).

### 4. Quality assessment

**Figure 2.**
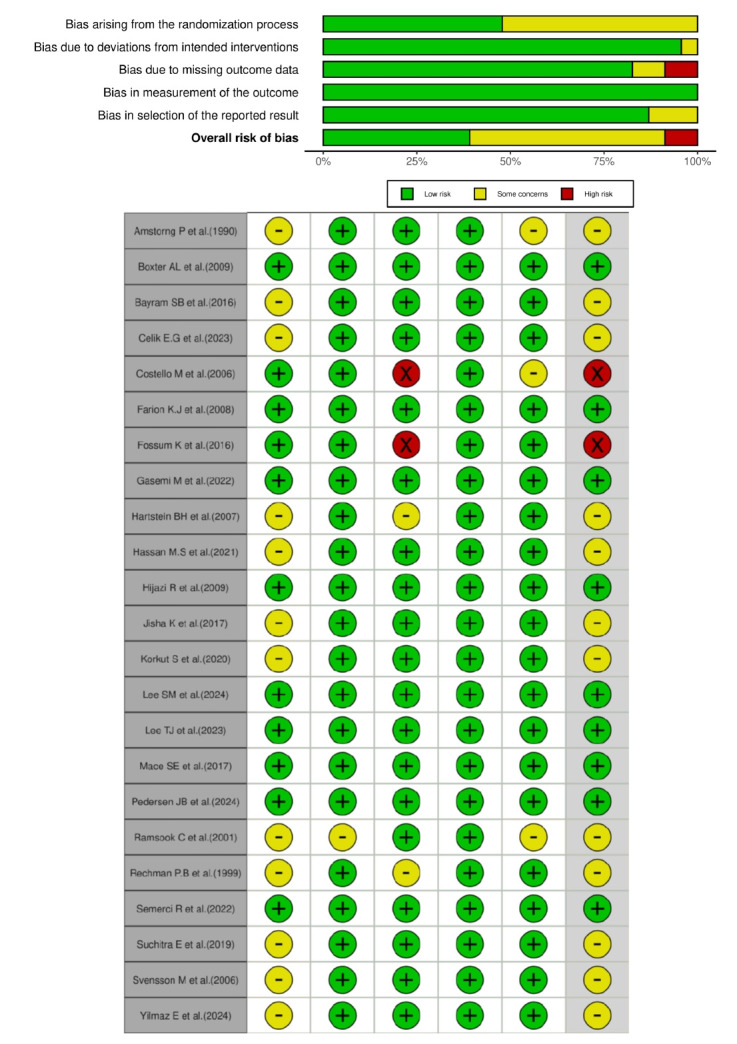
Risk of bias of the included studies.

The Cochrane Risk of Bias 2 tool [37] was utilized for quality assessment. This tool evaluates the risk of bias across five domains, including the randomization procedure, adherence to intended interventions, completeness of outcome data, accuracy of outcome measurement, and selective reporting of results [37,38]. Quality assessment was independently performed by two researchers. In cases of disagreement, the researchers engaged in thorough discussions, and if consensus could not be reached, the decision was finalized by consulting a third researcher (Figure 2).

### 5. Statistical analysis

The systematic analysis, synthesis, statistical integration, and reporting of results for the selected studies were conducted in accordance with the guidelines provided by Cochrane [39]. The meta-analysis of studies that applied thermotherapy and cryotherapy to measure pain and fear as outcome variables was performed using the Cochrane Review Manager software version 5.4 (The Cochrane Collaboration, London, UK). In this study, the characteristics of the participants and interventions (included first author, country, age, sex, IV needle gauge, intervention time, intervention temperature, main outcome measure, baseline outcome, post outcome, and author’s conclusion) were extracted. The primary outcome variable was pain measured during IV catheter insertion, and the effect size was calculated using the mean and SD, expressed as the standardized mean difference (SMD) 95% confidence interval (CI).

We followed the methodological guideline of the Higgins’ I² statistic to assess heterogeneity among studies. An I² value of 25% or lower was interpreted as low heterogeneity, 75% or higher as substantial heterogeneity, and values in between as moderate. In general, when the I² value exceeded 50%, a random-effects model was applied [40,41].

Subgroup analyses were planned to explore potential sources of heterogeneity in pain outcomes only, as fear outcomes were reported in too few studies to allow for meaningful subgroup analysis. The subgroup analyses were conducted based on intervention type (heat vs. cold), management methods (cold spray, other cold therapies, heat therapy), and participant age.

## RESULTS

### 1. Characteristics of included studies

In this study, 23 studies were reviewed to evaluate the effects of thermotherapy and cryotherapy on pain management during IV cannulation. Among these, 9 (39.1%) studies were conducted in North America (United States of America, 8; Canada, 1), 11 (47.8%) in Asia (India, 2; Korea, 2; Turkey, 5; Malaysia, 1; Iran, 1), and 3 (13.0%) in Europe and Oceania (Sweden, 1; Australia, 1; Denmark, 1). Regarding the target populations, 10 (43.5%) studies focused on adult participants, 7 (30.4%) on children, and 6 (26%) did not report participant age. Regarding needle gauges for IV cannulation, 18G needles were used in four (17.4%) studies, 20G needles in five (21.7%), < 22G in nine (39.1%), and unspecified gauge number or various gauges used in five (21.7%).

Among these studies, six multi-arm studies (A4, A12, A13, A14, A15, and A23) included two experimental groups and one control group. As a result, 29 comparisons were performed in the qualitative analysis. Of the 29 comparisons, in terms of interventions, 20 (66.6%) studies involved cryotherapy, including 13 (43.3%) utilizing sprays and 7 (24.1%) using ice cubes or gel packs. In contrast, nine (31.0%) studies employed heat therapy, including thermal energy equipment such as electric devices, bands, pillows, or pads, and hot packs. For the control groups, 12 (41.4%) studies used placebo interventions, while 17 (58.6%) provided no treatment as a comparator. From the 23 reviewed studies (29 comparisons in total), the final meta-analysis included 14 studies (19 comparisons) in which effect sizes were confirmed.

Table 1 summarizes the characteristics of all studies included in the qualitative synthesis. Studies without sufficient outcome data for effect size estimation were excluded from the final meta-analysis (14 studies, 19 comparisons). Appendix 2 provides a list of all studies reviewed in the systematic review.

### 2. The effect of heat and cold interventions on IV pain

The pooled overall effect size of the 14 studies that evaluated the impact of heat and cold interventions on IV pain was −0.85 (95% CI, −1.21 to −0.50), with a significant difference (Z = 4.73, *p* < .001). The heterogeneity test showed high heterogeneity among the studies (Higgins' I² = 86%), prompting the use of a random-effects model to analyze the effect size.

**Figure 3.**
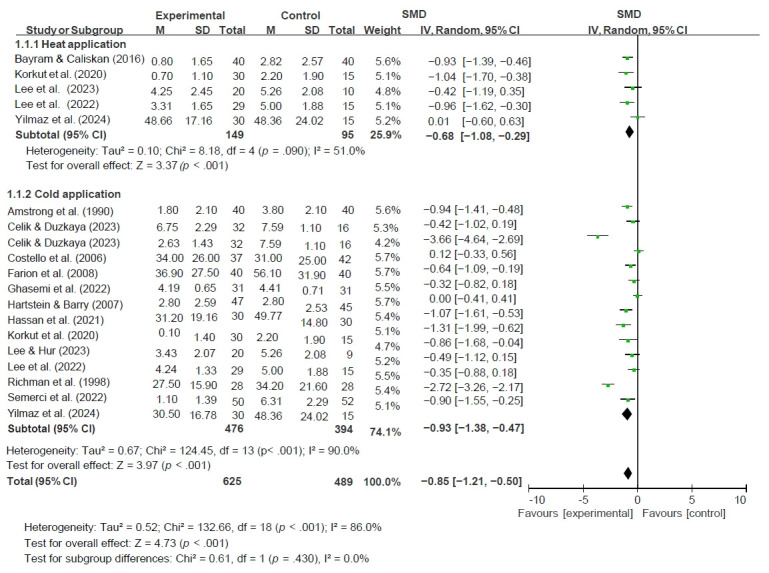
The effect of heat and cold intervention on intravenous pain. M = Mean; SD = Standard deviation; IV = Inverse variance; CI = Confidence interval; SMD = Standardized mean difference.

Subgroup analysis showed that heat interventions had an effect size [SMD (95% CI)] of −0.68 (−1.08 to −0.29), with Z = 3.37 (*p* < .001), and cold interventions had an effect size of -0.93 (−1.38 to −0.47), with Z = 3.97(*p* < .001). No significant difference was found between thermotherapy and cryotherapy (*p* = .430) (Figure 3).

### 3. Comparison of pain management methods

The subgroup analysis further explored the effects of different hot and cold therapy methods. The results showed that cold spray (SMD = −1.11; 95% CI, −1.94 to −0.28; Z = 2.63; *p* = .009), other cold therapies such as ice packs and electric bands (SMD = −0.75; 95% CI, −1.02 to −0.47; Z = 5.29; *p* < .001), and heat application (SMD = −0.68; 95% CI, −1.08 to −0.29; Z = 3.37; *p* < .001) all demonstrated significant effects. No significant differences were observed among the cold spray, other cold therapies, and heat therapy subgroups (*p* = .660), indicating comparable effectiveness among these interventions (Appendix 3).

### 4. Effectiveness of interventions based on age

The subgroup analysis was conducted to evaluate the effectiveness of interventions based on the participants' age groups. For children aged under 19 years, the interventions resulted in an SMD of −1.20 (95% CI, −2.06 to −0.35), with Z = 2.76 (*p* = .006). For adults aged 19 years or older, the interventions showed an SMD of −0.77 (95% CI, −1.03 to −0.51), with Z = 5.73 (*p* < .001). The group with no reported age showed an SMD of −0.41 (95% CI, −0.98 to 0.15), with Z = 1.45 (*p* = .150). There was no significant difference among the three groups (*p* = .290) (Appendix 4).

### 5. The effect of heat and cold interventions on IV-related fear

**Figure 4.**
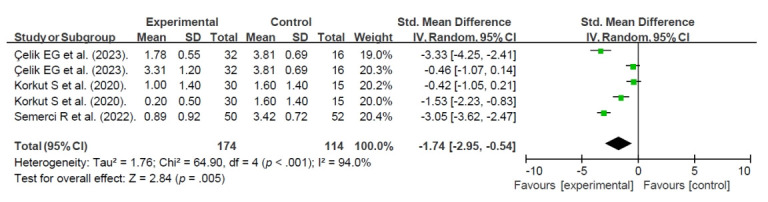
The effect of heat and cold intervention on intravenous-related fear. M = Mean; SD = Standard deviation; IV = Inverse variance; CI = Confidence interval; SMD = Standardized mean difference.

Of the 23 included studies, three reported measurable outcomes for fear. Of these, two studies were multi-arm designs, resulting in a total of five datasets analyzed. The pooled effect size SMD (95% CI) was −1.74 (−2.95 to −0.54), with a significant difference (Z = 2.84, *p* = .005). These findings indicate that both heat and cold interventions significantly reduce fear associated with IV procedures (Figure 4).

### 6. Publication bias

Of the 23 included studies in this systematic literature review, we assessed publication bias for 14 studies with available effect size estimates. The funnel plot shows that the studies are dispersed near the estimated effect size, with larger studies clustered at the top of the plot and smaller studies displaying greater variability, resulting in a wider distribution. The symmetrical shape of the funnel plot suggests no significant publication bias among the included studies (Appendix 5).

## DISCUSSION

We conducted a systematic review and meta-analysis to establish evidence on the effectiveness of thermotherapy and cryotherapy in alleviating pain and fear during IV cannulation. A total of 23 studies were included in the qualitative analysis, and 14 studies were selected for meta-analysis. The results showed that when comparing 625 participants in the intervention group and 489 in the control group, both thermotherapy and cryotherapy were effective in reducing pain (SMD = −0.85). The effect size of thermotherapy, expressed as SMD, was −0.68, while that of cryotherapy was −0.93, indicating that both interventions were effective in pain relief, with no significant difference between the two. According to Cohen's criteria, these effect sizes correspond to a large effect, suggesting that thermotherapy and cryotherapy are suitable non-pharmacological interventions for clinical application in pain management during IV cannulation [42]. Randomized controlled trials, systematic reviews, and meta-analyses conducted to date have aimed to determine the effectiveness of thermotherapy and cryotherapy. Primarily, studies examining the effectiveness of cold therapy have demonstrated its efficacy when applied in various forms, including vapor coolants [34], Buzzy^®^ device [20], and thermoelectric element tourniquets [31] Considering these findings along with the results of this meta-analysis, it can be concluded that cryotherapy is an effective method for alleviating pain during IV puncture. Various studies have examined temperature conditions of cryotherapy; in one study, the Coolsense device provided a temperature range of 12°C to −2°C for 3 to 5 seconds [23], and in another, a thermoelectric element tourniquet [31] provided a cooling temperature of 0~10°C for 10~30 seconds. Meanwhile, cold spray instantly evaporates upon contact with the skin, reducing the surrounding temperature to as low as −20°C. It is also used in minor surgical procedures requiring partial anesthesia. However, a major drawback of this method is that it can cause severe pain during the thawing process [43]. Cold spray shows significant effects even with an application time of approximately 10 seconds, but it has limitations regarding the areas of the skin where it can be applied [43-45].

These findings suggest that cold therapy is significantly effective in reducing pain during IV needle insertion when applied for less than one minute. In contrast, a study reported that applying cold therapy for 10 minutes did not alleviate pain and instead increased discomfort [46]. Considering this, cryotherapy appears to be most effective when applied for a short duration at temperatures below 10~12ºC during IV procedures. Therefore, careful consideration of the appropriate temperature and application time is necessary.

In this meta-analysis, in studies in which thermotherapy was applied, dry or moist heat, such as hot packs, moist towels, and electric pads, was applied at temperatures ranging from 40 to 60ºC [31,32]. Studies in which heat therapy was applied for 7 to 10 minutes demonstrated significant pain reduction, whereas studies with an application time of approximately one minute showed varying effects [31,32]. These findings suggest that, similar to cold therapy, the effectiveness of heat therapy may also be influenced by the duration of application.

Thermotherapy and cryotherapy are based on the gate control theory of pain [47,48]. Thermotherapy provides anesthetic and sedative effects by interfering with pain transmission. When applied locally, it increases blood flow and facilitates the removal of metabolic byproducts, thereby alleviating pain. Additionally, it promotes psychological relaxation, effectively reducing tension [49]. Cryotherapy, on the other hand, has been reported to potentially increase pain due to vasoconstriction [50]. However, it also reduces the sensitivity of nerve terminals, induces numbness, and provides immediate pain relief [47]. Based on this theoretical foundation, both thermotherapy and cryotherapy in this study appear to be effective in reducing pain during IV catheter insertion.

Pain is greatly influenced by subjective experiences [50]. In this study, the comparison of thermotherapy and cryotherapy showed that both methods are effective in alleviating pain. However, thermotherapy has been shown to shorten IV insertion time and increase patient satisfaction, whereas cryotherapy, despite reducing pain, has been associated with lower patient satisfaction [23]. Additionally, prolonged application of cryotherapy has been reported to cause discomfort and had no significant effect on pain relief [46]. Considering these findings, future research should not only explore various intervention methods but also allow individuals to choose between thermotherapy and cryotherapy based on their subjective preferences. This approach would help assess the impact of personal choice on psychological stability and pain relief outcomes.

In this study, the subgroup analysis by age demonstrated that the intervention was effective across all age groups. When applied to participants under the age of 19 years, the effect size was significant (SMD = −1.20; 95% CI, −2.06 to −0.35), as well as in adults aged ≥ 19 years (SMD = −0.80; 95% CI, −1.04 to −0.55). However, the effect size for the “no reported age” group was not significant (SMD = −0.41; 95% CI, −0.98 to 0.15). No significant difference was observed between the groups (*p* = .280). These findings suggest that thermotherapy and cryotherapy are effective in reducing pain associated with IV cannulation, regardless of age. However, rather than applying a generalized intervention, it is necessary to implement interventions that are specific to individual characteristics such as age and sex.

In this study, the effect of thermotherapy and cryotherapy on IV-related fear was analyzed based on three papers (five studies), revealing an overall effect size of −1.74 (−2.95 to −0.54). This finding suggests that thermotherapy and cryotherapy are not only effective in reducing pain but also in alleviating psychological stress associated with IV procedures.

Additionally, this study analyzed cold sprays as well as various cold therapy methods (e.g., gel bags, ice cubes) and compared the effects of thermotherapy and cryotherapy, clearly presenting their respective effectiveness. This analysis provides a more detailed examination of the clinical applicability of thermotherapy and cryotherapy and may contribute to proposing future implementation strategies. This study highlights that thermotherapy and cryotherapy are simple, non-invasive, and cost-effective methods for managing IV-related pain and fear. In particular, thermotherapy and cryotherapy can serve as valuable alternatives for pediatric patients, for whom medication use may be limited. While these interventions are generally safe, clinicians should be aware of potential adverse effects such as skin irritation, cold-induced discomfort, or thermal burns, depending on the temperature and duration of application. Therefore, appropriate safety measures and individualized precautions should be considered when implementing these methods in clinical settings.

The significance of this study is its distinct approach compared to previous research. Unlike prior studies, this study exclusively evaluated the pure effects of thermotherapy and cryotherapy by excluding mixed-intervention studies that incorporated medications or distraction techniques. Additionally, only studies with a control group were included in the analysis, ensuring a more rigorous assessment. Among the 23 included studies, 12 were rated as having some concerns, nine as low risk, and two as high risk in the overall quality assessment. The high proportion of studies with some concerns was mainly due to the inherent difficulty of blinding participants and personnel, given the nature of the interventions. In addition, studies that did not report outcome data sufficiently were assessed as high risk. Therefore, future research should aim to improve methodological rigor through better blinding procedures and more complete outcome reporting. Another limitation of this study is the challenge in providing sufficient evidence due to the limited number of studies applying only thermotherapy or cryotherapy during IV puncture. Additionally, the sub-analysis by age group lacked sufficient studies for each age category. Furthermore, the number of studies in which thermotherapy was applied during IV catheter insertion was limited, which restricts the generalizability of the results.

Therefore, we suggest that thermotherapy may have similar or even greater effects compared to cryotherapy, emphasizing the need for well-designed randomized controlled trials in the future. Future research should assess its effectiveness across various clinical settings, compare and analyze different tools and methods used in thermotherapy and cryotherapy, and propose an optimal intervention strategy.

## CONCLUSION

This systematic review and meta-analysis provides important evidence supporting the effectiveness of thermotherapy and cryotherapy in managing pain and fear associated with IV procedures. Both interventions showed large effect sizes in reducing pain and fear, with no significant difference between them. Given their non-invasive, cost-effective nature and applicability across age groups, thermotherapy and cryotherapy can be actively utilized in clinical practice, particularly for patients where pharmacological interventions are limited or less desirable. Future studies should further investigate their comparative effectiveness, optimal application conditions, and patient preferences to develop tailored pain management strategies.

## Figures and Tables

**Table 1.** Summary of the Effects of Heat or Cold Therapy on Pain during IV Cannula Insertion

Study	First author (year) Country	Age Sex (M/W)	I (No.)	IV size Intervention time Spraying distance Temperature	C (No.)	Main outcome measures	Baseline	Post test (M ± SD) (med [min~max])	Author's conclusion
A1	Armstrong et al.	NR	Vapocoolant spray	20G	No treatment	Pain	NR	I: 1.80±2.10	Ethyl chloride significantly reduced the pain of venipuncture
	(1990)		(ethyl chloride)	10 seconds	(40)			C: 3.80±2.10	
	United Kingdom	0/80	(40)	8 inches					
				N/A					
A2	Baxter et al.	I:26~55	Vapocoolant spray	22G	No treatment	Pain	NR	I: 1.1 [0~9.8]	Compared with no intervention, vapocoolant did not reduce pain significantly
	(2009)	C:26~55	(14)	5 seconds	(14)			C: 3.1 [0~9.9]	
	USA			5~10 cm		Fear			
		I:8/6		N/A				I:0.65 [0~4.5]	
		C:8/6						C: NR	
A3	Bayram & Caliskan	I:55.22±14.59	Moist heating pad	22G	No treatment	Pain	I: 1.05±1.58	I: 0.8±1.65	Local application of heat results in vasodilation, making intravenous catheter insertion easier and decreasing patients’ pain
	(2016)	C:54.0±10.23	(40)	10 minutes	(40)		C: 1.67±2.30	C: 2.82±2.57	
	Turkey			N/A					
		I:21/19		52°C (126°F)					
		C:25/15							
A4a	Celik & Duzkaya	I: 10.72±2.80	Cold spray	24G	No application	Pain	I: 7.19±2.74	I: 2.63±1.43	The pain and fear scores of the children in the control group were higher than the scores of those in the spray groups
	(2023)	C:11.22±2.74	(32)	5 seconds	(32)		C: 5.97±1.77	C: 7.59±1.10	
	Turkey			15 cm					
		I: 16/16		NR		Fear	I: 4.09±0.86	I: 1.78±0.55	
		C: 14/18					C: 2.91±1.06	C: 3.81±0.69	
A4b	Celik & Duzkaya	I: 10.06±2.99	Ice gel bag	24G	No application	Pain	I; 7.19±2.04	I: 6.75±2.29	The pain and fear scores of the children in the control group were higher than the scores of those in the ice groups
	(2023)	C:11.22±2.74	(32)	5 seconds	(32)		C: 5.97±1.77	C: 7.59±1.10	
	Turkey			15 cm					
		I: 21/11		NR		Fear	I: 3.72±0.85	I: 3.31±1.20	
		C: 14/18					C: 2.91±1.06	C: 3.81±0.69	
A5	Costello et al.	I: 12.7±2	Vapocoolant spray	22G	No application	Pain	NR	I: 34±26	Vapocoolant spray failed to measurably reduce pain associated with intravenous cannulation when compared to receiving no intervention
	(2006)	C: 13.2±3	(ethyl vinyl chloride)	5 seconds	(42)			C: 31±25	
	USA		(37)	NR					
		I: 15/22		N/A					
		C: 21/221							
A6	Farion et al.	I: 9.4±2.1	Vapocoolant spray	22~24G	Placebo spray (normal saline)	Pain	NR	I: 36.90±27.50	The use of vapocoolant spray significantly reduced pain compared to placebo
	(2008)	C:9.2±2.0	(1,1,1,3,3-pentaflouropropane	4~10 seconds	(40)			C: 56.10±31.90	
	Canada		and 1,1,1,2-tetrafluoroethane,)	8~18 cm					
		I:23/17	(40)	N/A					
		C:19/21							
A7	Fossum et al.	NR	Vapocoolant spray	20G	Placebo spray (sterile water)	Pain	NR	I: 2 [1~4]	Topical ethyl chloride was more effective than placebo in reducing pain during venous catheterization
	(2016)		(ethyl chloride)	5~8 seconds	(38)			C: 4 [2~5]	
	USA	NR	(38)	NR					
				36°F					
A8	Ghasemi et al.	NR	Vapocoolant spray	22G	No treatment	Pain	NR	I: 4.19±0.65	During and after the cannulation, the pain was significantly lower in the vapocoolant measurement
	(2022)		(31)	5 seconds	(31)			C: 4.41±0.71	than in control and music
	Iran	13/18		25 cm					
				N/A					
A9	Hartstein & Barry	NR	Vapocoolant spray(1,1,1,3,3-pentaflouropropaneand 1,1,1,2-tetrafluoroethane)	22~18G	No application	Pain	NR	I: 2.80±2.59	This study reported no significant difference in pain perception with the use of skin coolant prior to IV placement
	(2007)		(47)	2~4 seconds	(45)			C: 2.80±2.53	
	USA	NR		7~12 cm					
				36°F					
A10	Hassan et al.	I:9.57±1.49	Ice compression	22G	Without any analgesics	Pain	NR	I: 31.2±19.16	Ice compression is an effective intervention to reduce pain during venipuncture and IV cannulation among
	(2021)	C:9.20±1.45	(ice cube)	2 minutes	(30)			C: 49.77±14.80	primary school children
	Malaysia		(30)	N/A					
		I:19/11		N/A					
		C:20/10							
A11	Hijazi et al.	I: 59.9±19.0	Vapocoolant spray	22~18G	Placebo spray (pure water)	Pain	NR	I: 12 [5~40]	Topical alkane vapocoolant spray is effective, acceptable, and safe in reducing pain with peripheral intravenous cannulation in adult
	(2009)	C: 56.3±20.0	(propane, butane, and pentane blend)	2 seconds	(98)			C: 36 [29~51]	
	Australia		(103)	12 cm					
		I: 57/46		N/A					
		C: 52/46							
A12a	Jisha et al.	NR	Dry heat application	NR	Placebo spray (sterile water)	Pain	NR	I: 1 [NR]	Dry heat applications significantly reduced pain compared to the control group
	(2017)			7 minutes				C: 7 [NR]	
	India	NR		N/A					
				120~140°F					
A12b	Jisha et al.	NR	Moist heat application (wrapping a moist towel)	NR	NR	Pain	NR	I: 5 [NR]	Moist heat applications significantly reduced pain compared to the control group
	(2017)			7 minutes				C: 7 [NR]	
	India	NR		N/A					
				110~115°F					
A13a	Korkut et al.	I: 48.8±14.6	Cold pack	20G	No applications	Pain	I: 0.1±0.5	I: 1.1±1.4	Applying local cold application before IV insertion reduced both pain and anxiety levels of the patients
	(2020)	C: 30.23±7.8	(30)	60 seconds	(30)		C: 0±0	C: 2.2±1.9	
	Turkey			N/A					
		I: 22/8		NR		Fear	I:0.6±1.1	I: 1.0±1.4	
		C:24/6					C:0.3±0.8	C: 1.6±1.4	
A13b	Korkut et al.	I: 56±7.5	Hot pack	20G	No applications	Pain	I: 0.1±0.4	I: 0.7±1.1	Applying local hot application before IV insertion reduced both pain and anxiety levels of the patients
	(2020)	C: 30.23±7.8	(30)	60 seconds	(30)		C: 0±0	C: 2.2±1.9	
	Turkey			N/A					
		I: 21/9		NR		Fear	I:0.6±1.1	I: 0.2±0.5	
		C:24/6					C:0.3±0.8	C: 1.6±1.4	
A14a	Lee & Hur	I:45.9±13.4	Warm electric pad	18G	Placebo application	Pain	I: 0.00	I:4.25±2.45	The thermoelectric tourniquet has no significant effect on relieving intravenous injection pain
	(2023)	C:49.8±14.4	(20)	10~30 seconds	(19)		C: 0.00	C:5.26±2.08	
	Korea			NA					
		I:8/12		40~45°C					
		C:7/12							
A14b	Lee & Hur	I:47.6±13.2	Cold electric pad	18G	Placebo application	Pain	I:0.08±0.34	I: 3.43±2.07	The thermoelectric tourniquet has the effect of relieving intravenous injection pain in cold therapy
	(2023)	C:49.8±14.4	(20)	10~30 seconds	(19)		C:5.3±2.1	C: 5.26±2.08	
	Korea			NA					
		I:7/13		0~10°C					
		C:7/12							
A15a	Lee et al.	I:51.8±14.6	Warm electric pad	18G	Placebo application	Pain	NR	I: 3.31± 1.65	Heat therapy applied with thermoelectric tourniquet during venipuncture exerted pain-relief and stress-reduction effects
	(2022)	C:48.4±12.5	(29)	5~60 seconds	(30)			C: 5.00± 1.88	
	Korea			NA					
		I:9/20		40~45°C					
		C:13/17							
A15b	Lee et al.	I:45.5±14.0	Cold electric pad	18G	Placebo application	Pain	NR	I: 4.24± 1.33	Cold therapy applied with thermoelectric tourniquet during venipuncture exerted pain-relief and stress-reduction effects
	(2022)	C:48.4±12.5	(29)	5~60 seconds	(30)			C:5.00± 1.88	
	Korea			NA					
		I:15/14		0~10°C					
		C:13/17							
A16	Mace et al.	I: 43.0±16.6	Vapocoolant spray (1,1,1,3,3-pentaflouropropan e and 1,1,1,2-tetra fluoroethane)	18~22G	Placebo spray (sterile water)	Pain	NR	I: 1 [0~3]	Vapocoolant spray was effective in significantly relieving the pain of PIV cannulation in adults
	(2017)	C:42.7±16.6	(150)	4~10 seconds	(150)			C: 3 [1.2~5]	
	USA			3~7 inches					
		I: 56/94		N/A					
		C:64/86							
A17	Pedersen et al.	I: 61	Cryospray	20G	Placebo spray (normal saline)	Pain	I: 0 [0~0]	I: 1 [0~3]	Patients reported lower pain scores and a greater preference for cryospray in future procedures
	(2024)	C:63.5	(Chloraethyl spray)	4 seconds	(64)		C: 0 [0~2.5]	C: 3 [2~5]	
	Denmark		(64)	(2 times, 2 seconds)					
		I:33/31		15~20 cm					
		C:35/29		N/A					
A18	Ramsook et al.	I: 9	Vapocoolant spray	NR	Placebo spray (alcohol)	Pain	NR	I: 4 [NR]	Vapocoolant spray
	(2001)	C: 10	(ethyl chloride)	5 seconds	(108)			C: 6 [NR]	showed no benefit in reducing IV cannula insertion pain
	USA		(114)	6 inches					
		I: 50/64		N/A					
		C: 51/57							
A19	Richman et al.	I: 8.50±2.27	Ice pack	18G	No treatment	Pain	NR	I: 27.50±15.90	Pretreatment with ice did not reduce overall
	(1999)	C:8.33±2.12	(28)	10 minutes	(28)			C:34.20±21.60	mean pain scores from IV catheter insertion
	USA			N/A					
		I: 25/25		NR					
		C: 28/24							
A20	Semerci et al.	I: 8.50±2.27	Cold spray	24G	Routine practice	Pain	NR	I: 1.10±1.39	Cold spray was more effective than standard care in reducing the
	(2022)	C:8.33±2.12	(chloromethyl ethyl spray)	5 seconds	(52)			C:6.31±2.29	level of pain, and fear in children ages 5~12 years during venipuncture
	Turkey		(50)	15 cm					
		I: 25/25		N/A		Fear		I: 0.89±0.92	
		C: 28/24						C: 3.42±0.72	
A21	Suchitra et al.	NR	Electric heating pad	24~26G	No heat application	Pain	NR	Only categorical variable reported	Dry heat before IV insertion improves venipuncture ease and reduces perceived pain
	(2019)		(42)	10 minutes	(42)				
	India	I: 22/20		N/A					
		C: 20/22		40°C					
A22	Svensson et al.	I: 49	Local warming pillow	18G	No warming	Pain	NR	I: 1.74	Local warming before peripheral IV insertion appears not to have any pain reducing effect
	(2006)	C: 49	(61)	60 seconds	(64)			[0.1~4.8]	
	Sweden			N/A				C: 2.01	
		I: 31/30		39~40°C				[0.3~7.2]	
		C: 40/24							
A23a	Yilmaz & Yilmaz	I: 53.20±11.15	Cold gel pack application	20G	No treatment	Pain	NR	I: 30.50± 16.78	One-minute local cold application reduced pain during peripheral IV catheterization
	(2024)	C:53.10±13.51	(30)	1 minute	(30)			C: 48.36±24.02	
	Turkey			N/A					
				NR					
A23b	Yilmaz & Yilmaz	I: 53.20±11.15	Dry hot pack application	20G	No treatment	Pain	NR	I: 48.66 ±17.16	One-minute local warm application reduced pain during peripheral IV catheterization
	(2024)	C:53.10±13.51	(30)	1 minute	(30)			C: 48.36±24.02	
	Turkey			N/A					
				40~42°C					

NR = Not reported; N/A = Not applicable; M = Men; W = Women; I = Intervention group; C = Control group; IV = Intravenous; G = Gauze; M = Mean; SD = Standard deviation; Med = Median; min = Minimum; max = Maximum.

## Data Availability

The data that support the findings of this study are available from the corresponding author upon reasonable request.

## Appendix 1. Literature Search Strategy

**Table:**

**1. PubMed: 1212 Results **
#1(("phlebotomy"[MeSH Terms] OR "phlebotomy"[All Fields] OR "venipuncture"[All Fields] OR "venipunctures"[All Fields] OR ("injections, intravenous"[MeSH Terms] OR ("injections"[All Fields] AND "intravenous"[All Fields]) OR "intravenous injections"[All Fields] OR "injections intravenous"[All Fields]) OR "iv"[All Fields]) OR (("intraveneous"[All Fields] OR "intraveneously"[All Fields] OR "intravenous"[All Fields] OR "intravenously"[All Fields]) AND ("catheter s"[All Fields] OR "catheters"[MeSH Terms] OR "catheters"[All Fields] OR "catheter"[All Fields])) OR (("intraveneous"[All Fields] OR "intraveneously"[All Fields] OR "intravenous"[All Fields] OR "intravenously"[All Fields]) AND ("canulated"[All Fields] OR "canulation"[All Fields] OR "canule"[All Fields])) OR (("intraveneous"[All Fields] OR "intraveneously"[All Fields] OR "intravenous"[All Fields] OR "intravenously"[All Fields]) AND ("cannula"[MeSH Terms] OR "cannula"[All Fields] OR "cannulae"[All Fields] OR "cannulas"[All Fields] OR "cannula s"[All Fields]))
#2 (("hot"[All Fields] OR "warm"[All Fields] OR ("hot temperature"[MeSH Terms] OR ("hot"[All Fields] AND "temperature"[All Fields]) OR "hot temperature"[All Fields] OR "heat"[All Fields]) OR ("ice"[MeSH Terms] OR "ice"[All Fields]) OR ("common cold"[MeSH Terms] OR ("common"[All Fields] AND "cold"[All Fields]) OR "common cold"[All Fields] OR "cold"[All Fields] OR "cold temperature"[MeSH Terms] OR ("cold"[All Fields] AND "temperature"[All Fields]) OR "cold temperature"[All Fields]) OR ("vapocoolant"[All Fields] OR "vapocoolants"[All Fields]) OR ("cryotherapy"[MeSH Terms] OR "cryotherapy"[All Fields] OR "cryotherapies"[All Fields]) OR ("hyperthermia, induced"[MeSH Terms] OR ("hyperthermia"[All Fields] AND "induced"[All Fields]) OR "induced hyperthermia"[All Fields] OR "thermotherapy"[All Fields] OR "thermotherapies"[All Fields]))
#3 (("pain"[MeSH Terms] OR "pain"[All Fields] OR ("fear"[MeSH Terms] OR "fear"[All Fields]))
#4 #1 AND #2 AND #3
**2. Embase: 4066 Results**
#1 ('venipuncture'/exp OR venipuncture OR 'injections, intravenous'/exp OR 'injections, intravenous' OR (('injections,'/exp OR injections,) AND intravenous) OR iv OR 'intravenous catheter'/exp OR 'intravenous catheter' OR (intravenous AND ('catheter'/exp OR catheter)) OR 'intravenous canulation' OR (intravenous AND canulation) OR 'intravenous cannula'/exp OR 'intravenous cannula' OR (intravenous AND ('cannula'/exp OR cannula)))
#2 (hot OR warm OR 'heat'/exp OR heat OR 'ice'/exp OR ice OR 'cold'/exp OR cold OR 'vapocoolant'/exp OR vapocoolant OR 'cryotherapy'/exp OR cryotherapy OR 'thermotherapy'/exp OR thermotherapy)
('pain'/exp OR pain OR 'fear'/exp OR fear)
#4 #1 AND #2 AND #3
**3. Cochrane: 1159 Results**
(((venipuncture) OR (Injections, intravenous) OR (IV) OR (intravenous catheter) OR (intravenous canulation) OR (intravenous cannula)) AND ((Pain) OR (fear)) AND ((hot) OR (warm) OR (heat) OR (ice) OR (cold) OR (vapocoolant) OR (cryotherapy) OR (thermotherapy))) in Title Abstract Keyword
**4. CINAHL: 49 Results**
((venipuncture) OR (Injections, intravenous) OR (IV) OR (intravenous catheter) OR (intravenous canulation) OR (intravenous cannula)) AND ((Pain) OR (fear)) AND ((hot) OR (warm) OR (heat) OR (ice) OR (cold) OR (vapocoolant) OR (cryotherapy) OR (thermotherapy)))

## Appendix 2. Studies Included in Systematic Review and Meta-analysis

A1. Armstrong P, Young C, McKeown D. Ethyl chloride and venepuncture pain: a comparison with intradermal lidocaine. Canadian Journal of Anesthesia. 1990;37(6):656–658. https://doi.org/10.1007/BF03006485

A2. Baxter AL, Leong T, Mathew B. External thermomechanical stimulation versus vapocoolant for adult venipuncture pain: pilot data on a novel device. The Clinical Journal of Pain. 2009;25(8):705–710. https://doi.org/10.1097/AJP.0b013e3181af1236

A3. Bayram SB, Caliskan N. Effects of local heat application before intravenous catheter insertion in chemotherapy patients. Journal of Clinical Nursing. 2016;25(11–12):1740–1747. https://doi.org/10.1111/jocn.13193

A4. Celik EG, Duzkaya DS. The impact of cold spray and ice application during intravenous access on pain and fear in children aged 7–15 years in the pediatric emergency unit: a randomized controlled trial. Journal of Emergency Nursing. 2024;50(2):264–272. https://doi.org/10.1016/j.jen.2023.11.012

A5. Costello M, Ramundo M, Christopher NC, Powell KR. Ethyl vinyl chloride vapocoolant spray fails to decrease pain associated with intravenous cannulation in children. Clin Pediatrics. 2006;45(7):628–632. https://doi.org/10.1177/0009922806291013

A6. Farion KJ, Splinter KL, Newhook K, Gaboury I, Splinter WM. The effect of vapocoolant spray on pain due to intravenous cannulation in children: a randomized controlled trial. Canadian Medical Association Journal. 2008;179(1):31–36. https://doi.org/10.1503/cmaj.070874

A7. Fossum K, Love SL, April MD. Topical ethyl chloride to reduce pain associated with venous catheterization: a randomized crossover trial. The American Journal of Emergency Medicine. 2016;34(5):845–850. https://doi.org/10.1016/j.ajem.2016.01.039

A8. Ghasemi M, Hoseinialiabadi P, Yazdanpanah F, Mahani MA, Malekyan L, Najafi K, et al. Comparison of music and vapocoolant spray in reducing the pain of venous cannulation in children age 6-12: a randomized clinical trial. BMC Pediatrics. 2022;22(1):237. https://doi.org/10.1186/s12887-022-03271-9

A9. Hartstein BH, Barry JD. Mitigation of pain during intravenous catheter placement using a topical skin coolant in the emergency department. Emergency Medicine Journal. 2008;25(5):257–261. https://doi.org/10.1136/emj.2006.044776

A10. Hassan MS, Fauzi MH, MdNoh AY, Abdullah AA, Nor JM, Yaacob N, et al. Effectiveness of ice compression to reduce pain among primary school children venipuncture and peripheral intravenous cannulation in emergency department north-eastern Malaysia. International Medical Journal. 2022;29(1):34–37.

A11. Hijazi R, Taylor D, Richardson J. Effect of topical alkane vapocoolant spray on pain with intravenous cannulation in patients in emergency departments: randomised double blind placebo controlled trial. BMJ. 2009;338:b215. https://doi.org/10.1136/bmj.b215

A12. Jisha K, Latha S, Joseph G. A comparative study on impact of dry versus moist heat application on feasibility of peripheral intravenous cannulation among the patients of a selected hospital at Mangalore. Journal of Health and Allied Sciences NU. 2017;7(3):21–24. https://doi.org/10.1055/s-0040-1708719

A13. Korkut S, Karadağ S, Doğan Z. The effectiveness of local hot and cold applications on peripheral intravenous catheterization: a randomized controlled trial. Journal of PeriAnesthesia Nursing. 2020;35(6):597–602. https://doi.org/10.1016/j.jopan.2020.04.011

A14. Lee SM, Hur MH. Thermoelectric tourniquet-assisted thermotherapy and cryotherapy for pain, regional blood flow, and satisfaction with intravenous injections among hospitalized patients in Korea: a randomized controlled trial. Journal of Korean Biological Nursing Science. 2024;26(4):323-336. https://doi.org/10.7586/jkbns.24.027

A15. Lee TJ, Her J, Hur MH. Effects of a thermoelectric element tourniquet on venipuncture pain and stress relief in Korea: a randomized controlled trial. Journal of Korean Biological Nursing Science. 2025;27(2):179-190. https://doi.org/10.7586/jkbns.25.018

A16. Mace SE. Prospective, randomized, double-blind controlled trial comparing vapocoolant spray vs placebo spray in adults undergoing venipuncture. The American Journal of Emergency Medicine. 2016;34(5):798–804. https://doi.org/10.1016/j.ajem.2016.01.002

A17. Pedersen JB, Martensen A, Funder P, Bakke SA, Bhavsar RP, Strom T. Cryospray reduces pain during venous cannulation in elective surgery patients: a randomized placebo-controlled study. BMC Anesthesiology. 2024;24(1):466. https://doi.org/10.1186/s12871-024-02858-2

A18. Ramsook C, Kozinetz CA, Moro-Sutherland D. Efficacy of ethyl chloride as a local anesthetic for venipuncture and intravenous cannula insertion in a pediatric emergency department. Pediatric Emergency Care. 2001;17(5):341–343. https://doi.org/10.1097/00006565-200110000-00005

A19. Richman PB, Singer AJ, Flanagan M, Thode Jr HC. The effectiveness of ice as a topical anesthetic for the insertion of intravenous catheters. The American Journal of Emergency Medicine. 1999;17(3):255–257. https://doi.org/10.1016/S0735-6757(99)90119-5

A20. Semerci R, Akarsu O, Kilic D. The effect of Buzzy and cold spray on pain, anxiety, and fear of children during venipuncture in pediatric emergency department in Turkey: a randomized controlled study. Journal of Pediatric Nursing. 2023;68:e1–e7. https://doi.org/10.1016/j.pedn.2022.08.019

A21. Suchitra E, Srinivasan R. Effectiveness of dry heat application on ease of venepuncture in children with difficult intravenous access: a randomized controlled trial. Journal of Specialists in Pediatric Nursing. 2020;25(1):e12273. https://doi.org/10.1111/jspn.12273

A22. Svensson M, Rosén S, Nilsson U. Local warming to reduce pain on peripheral intravenous cannula insertion: a randomised controlled study. Journal of Advanced Perioperative Care. 2006;2(3):107–112.

A23. Yilmaz E, Yılmaz D. The effect of three different nonpharmacological methods on cannulation success during peripheral intravenous catheter placement in the emergency unit: a randomized controlled trial. BMC Anesthesiology. 2024;24(1):362. https://doi.org/10.1186/s12871-024-02723-2

## References

[1] Alexandrou E, Ray-Barruel G, Carr PJ, Marsh N, Frost S, Inwood S (2015). International prevalence of the use of peripheral intravenous catheters. Journal of Hospital Medicine.

[2] Berger S, Winchester K, Principe RB, Culverwell E (2022). Prevalence of peripheral intravenous catheters and policy adherence: a point prevalence in a tertiary care university hospital. Journal of Clinical Nursing.

[3] Brady T, Bruno F, Marchionni C, Paquet F (2016). Prevalence and maintenance practices of peripheral intravenous catheters. Vascular Access.

[4] Thomas JG, Spitalnick JS, Hadley W, Bond DS, Wing RR (2014). Development of and feedback on a fully automated virtual reality system for online training in weight management skills. Journal of Diabetes Science and Technology.

[5] Heden L, von Essen L, Ljungman G (2020). Children's self‐reports of fear and pain levels during needle procedures. Nursing Open.

[6] Sorensen K, Skirbekk H, Kvarstein G, Woien H (2020). Children’s fear of needle injections: a qualitative study of training sessions for children with rheumatic diseases before home administration. Pediatric Rheumatology.

[7] Gilchrist PT, Thijsen A, Masser BM, France CR, Davison TE (2021). Improving the donation experience and reducing venipuncture pain by addressing fears among whole‐blood and plasma donors. Transfusion.

[8] Campos LB, Martins JR, Arreguy-Sena C, Alves MdS, Teixeira CV, Souza LCd (2016). Experiences of hospitalized patients with the venipuncture process. Escola Anna Nery.

[9] Duncanson E, Le Leu RK, Shanahan L, Macauley L, Bennett PN, Weichula R (2021). The prevalence and evidence-based management of needle fear in adults with chronic disease: a scoping review. Plos One.

[10] Cozzi G, Valerio P, Kennedy R (2021). A narrative review with practical advice on how to decrease pain and distress during venepuncture and peripheral intravenous cannulation. Acta Paediatrica.

[11] Pathan A, Shetty Narasimha K, Naik A, Ranade A (2024). A pilot open-label randomized study to evaluate the safety, tolerability, and acceptability of the IntegriMedical^®^ needle free injection system versus a conventional needle-based system in healthy volunteers, using normal saline as a placebo. Medical Devices: Evidence and Research.

[12] Belevcikli M, Altan H, Demir O (2023). Effect of the new needle-free injection system on pain perception and dental anxiety during anesthesia: randomized controlled split-mouth study. Journal of Dental Anesthesia and Pain Medicine.

[13] Umemura H, Takahashi H, Fukuda Y, Soma H, Aoki R, Takei N (2024). Use of finer needles for venipuncture increases in vitro haemolysis despite reducing persistent pain and nerve injury: a retrospective study. Annals of Clinical Biochemistry.

[14] Goudra BG, Galvin E, Singh PM, Lions J (2014). Effect of site selection on pain of intravenous cannula insertion: a prospective randomised study. Indian Journal of Anaesthesia.

[15] McGowan D (2014). Peripheral intravenous cannulation: managing distress and anxiety. British Journal of Nursing.

[16] McLenon J, Rogers MA (2019). The fear of needles: a systematic review and meta‐analysis. Journal of Advanced Nursing.

[17] Cooke M, Ullman AJ, Ray-Barruel G, Wallis M, Corley A, Rickard CM (2018). Not “just” an intravenous line: consumer perspectives on peripheral intravenous cannulation (PIVC). An international cross-sectional survey of 25 countries. Plos One.

[18] Carr PJ, Rippey JC, Cooke ML, Trevenen ML, Higgins NS, Foale AS (2019). Factors associated with peripheral intravenous cannulation first-time insertion success in the emergency department. a multicentre prospective cohort analysis of patient, clinician and product characteristics. BMJ Open.

[19] Thybo KH, Friis SM, Aagaard G, Jensen CS, Dyekjær CD, Jorgensen CH (2022). A randomized controlled trial on virtual reality distraction during venous cannulation in young children. Acta Anaesthesiologica Scandinavica.

[20] Bourdier S, Khelif N, Velasquez M, Usclade A, Rochette E, Pereira B (2021). Cold vibration (buzzy) versus anesthetic patch (EMLA) for pain prevention during cannulation in children: a randomized trial. Pediatric Emergency Care.

[21] Xess PA, Sarna R, Sethi S, Chauhan R, Meena SC, Saini V (2024). Effect of CoolSense and EMLA cream on pain during intravenous cannulation in pediatric population: a randomized, controlled trial. Indian Journal of Pediatrics.

[22] Ghasemi M, Hoseinialiabadi P, Yazdanpanah F, Mahani MA, Malekyan L, Najafi K (2022). Comparison of music and vapocoolant spray in reducing the pain of venous cannulation in children age 6-12: a randomized clinical trial. BMC Pediatrics.

[23] Korkut S, Karadag S, Dogan Z (2020). The effectiveness of local hot and cold applications on peripheral intravenous catheterization: a randomized controlled trial. Journal of PeriAnesthesia Nursing.

[24] Choi SH, Baek KH, Lee JY, Lim HB, Lee JY, Yoon S (2006). The effect of warm and ice application for pain control caused by arteriovenous fistula needling under hemodialysis. Journal of Korean Clinical Nursing Research.

[25] Lane E, Latham T (2009). Managing pain using heat and cold therapy. Paediatric Nursing.

[26] Melzack R, Wall PD, Steptoe A, Wardle J (1994). Psychosocial processes and health: a reader.

[27] Erdogan B, Aytekin Ozdemir A (2021). The effect of three different methods on venipuncture pain and anxiety in children: distraction cards, virtual reality, and Buzzy^®^ (randomized controlled trial). Journal of Pediatric Nursing.

[28] Mohamed HG, Mohamed MAF (2019). Effect of local heat application on complaints of patients with moderate knee osteoarthritis. American Journal of Nursing Research.

[30] Halm M, Lindquist R, Lindquist R, Tracy MF, Snyder M (2022). Complementary therapies in nursing: promoting integrative care.

[31] Lee SM, Hur MH (2024). Thermoelectric tourniquet-assisted thermotherapy and cryotherapy for pain, regional blood flow, and satisfaction with intravenous injections among hospitalized patients in Korea: a randomized controlled trial. Journal of Korean Biological Nursing Science.

[32] Hur MH, Choi HS (2021). Effects of a thermoelectric element band on venipuncture-associated pain and anxiety: a randomized controlled trial. Asian Nursing Research.

[33] Wang L, Fang L, Zhou Y, Fang X, Liu J, Qu G (2023). Efficacy and safety of vapocoolant spray for vascular puncture in children and adults: a systematic review and meta-analysis. Plos One.

[34] Griffith RJ, Jordan V, Herd D, Reed PW, Dalziel SR (2016). Vapocoolants (cold spray) for pain treatment during intravenous cannulation. Cochrane Database of Systematic Reviews.

[35] Jin F, Wang X, Qi M, Zhang W, Zhang Y (2024). Effectiveness and safety of Buzzy device in needle-related procedures for children under twelve years of age: a systematic review and meta-analysis. Medicine.

[36] Page MJ, McKenzie JE, Bossuyt PM, Boutron I, Hoffmann TC, Mulrow CD (2021). The PRISMA 2020 statement: an updated guideline for reporting systematic reviews. BMJ.

[37] Sterne JA, Savović J, Page MJ, Elbers RG, Blencowe NS, Boutron I (2019). RoB 2: a revised tool for assessing risk of bias in randomised trials. BMJ.

[38] Kim SY, Park DA, Seo HJ, Shin SS, Lee SJ, Jang BH (2021). NECA’s guidance for assessing tools of risk of bias.

[39] Haddaway NR, Page MJ, Pritchard CC, McGuinness LA (2022). PRISMA2020: an R package and Shiny app for producing PRISMA 2020‐compliant flow diagrams, with interactivity for optimised digital transparency and Open Synthesis. Campbell Systematic Reviews.

[40] Higgins J, Thomas J (2011). Cochrane handbook for systematic reviews of interventions (current version) [Internet]. https://training.cochrane.org/handbook/current.

[41] Cho JH (2020). Theory and practice of meta-analysis. Journal of Rhinology.

[42] Goulet-Pelletier JC, Cousineau D (2018). A review of effect sizes and their confidence intervals, part I: the Cohen’s d family. The Quantitative Methods for Psychology.

[43] Armstrong P, Young C, McKeown D (1990). Ethyl chloride and venepuncture pain: a comparison with intradermal lidocaine. Canadian Journal of Anesthesia.

[44] Fossum K, Love SL, April MD (2016). Topical ethyl chloride to reduce pain associated with venous catheterization: a randomized crossover trial. The American Journal of Emergency Medicine.

[45] Farion KJ, Splinter KL, Newhook K, Gaboury I, Splinter WM (2008). The effect of vapocoolant spray on pain due to intravenous cannulation in children: a randomized controlled trial. Canadian Medical Association Journal.

[46] Richman PB, Singer AJ, Flanagan M, Thode HC (1999). The effectiveness of ice as a topical anesthetic for the insertion of intravenous catheters. The American Journal of Emergency Medicine.

[47] Sheppard M, Wright MW (2006). Principles and practice of high dependency nursing.

[48] Melzack R (1996). Gate control theory: on the evolution of pain concepts. Pain Forum.

[49] Foulkes T, Wood J (2007). Mechanisms of cold pain. Channels.

[50] McGrath PA (1994). Psychological aspects of pain perception. Archives of Oral Biology.

